# Predictive models of minimal hepatic encephalopathy for cirrhotic patients based on large-scale brain intrinsic connectivity networks

**DOI:** 10.1038/s41598-017-11196-y

**Published:** 2017-09-14

**Authors:** Yun Jiao, Xun-Heng Wang, Rong Chen, Tian-Yu Tang, Xi-Qi Zhu, Gao-Jun Teng

**Affiliations:** 1 Jiangsu Key Laboratory of Molecular and Functional Imaging, Department of Radiology, Zhongda Hospital, Medical School of Southeast University, Nanjing, 210009 China; 2 College of Life Information Science and Instrument Engineering, Hangzhou Dianzi University, Hangzhou, 310018 China; 3 Department of Diagnostic Radiology and Nuclear Medicine, University of Maryland, School of Medicine, Baltimore, MD 21201 USA; 4 Department of Radiology, The Second Hospital of Nanjing, Medical School of Southeast University, Nanjing, 210003 China

## Abstract

We aimed to find the most representative connectivity patterns for minimal hepatic encephalopathy (MHE) using large-scale intrinsic connectivity networks (ICNs) and machine learning methods. Resting-state fMRI was administered to 33 cirrhotic patients with MHE and 43 cirrhotic patients without MHE (NMHE). The connectivity maps of 20 ICNs for each participant were obtained by dual regression. A Bayesian machine learning technique, called Graphical Model-based Multivariate Analysis, was applied to determine ICN regions that characterized group differences. The most representative ICNs were evaluated by the performance of three machine learning methods (support vector machines (SVMs), multilayer perceptrons (MLP), and C4.5). The clinical significance of these potential biomarkers was further tested. The temporal lobe network (TLN), and subcortical network (SCN), and sensorimotor network (SMN) were selected as representative ICNs. The distinct functional integration patterns of the representative ICNs were significantly correlated with behavior criteria and Child-Pugh scores. Our findings suggest the representative ICNs based on GAMMA can distinguish MHE from NMHE and provide supplementary information to current MHE diagnostic criteria.

## Introduction

Approximately 33–50% of patients with liver cirrhosis without clinical symptoms of encephalopathy have minimal hepatic encephalopathy (MHE)^1^. MHE can result in a wide spectrum of neuropsychiatric and neurophysiological impairments^2, 3^. Patients with MHE show deficits in several cognitive domains, such as attention, working memory, motor, visual perception, visuoconstruction and motor skills^3–6^.

MHE reduces quality of life, impairs daily living, and decreases life span^1, 3, 7–9^. Previous studies^3, 10^ suggest that early accurate diagnosis of MHE can prevent the development of overt hepatic encephalopathy (HE) and has a positive impact on patients and their survival. However, MHE is mainly diagnosed based on neuropsychological/neurophysiological tests and is often undiagnosed^11^ because 1) the diagnostic criteria for MHE have not been standardized, and 2) in many patients there is no overt clinical evidence of impaired cognition^3^.

Resting-state functional magnetic resonance imaging (rs-fMRI) can probe cerebral intrinsic functional architecture and measure spontaneous neuronal activity at a baseline state^12^, which is a useful technique for investigating the neuropathological mechanisms underlying functional deficits in MHE^13–18^. Brain activity at rest can be spatially organized in a set of large-scale coherent patterns called intrinsic connectivity networks (ICNs), which are suggested to represent inherent patterns of specific brain functions^19^. ICNs with variations that have been correlated with MHE are the default mode network (DMN), dorsal attention network (DAN), visual network (VN), and auditory network (AN)^20–23^.

Given the rs-fMRI and related ICN findings described above, ICN-based diagnostic models may hold the promise of enhancing or complementing behavioral assessment for MHE. Previous studies have indicated that the benefits of MRI/fMRI-based diagnostic models are twofold^24–26^. First, these methods could provide objective and accurate neuroimaging markers for subsequent investigation. Second, these MR-based methods may provide information that is complementary to that obtained by behavioral assessment. Therefore, several studies have aimed to discriminate MHE from NMHE based on ICNs. Qi *et al*. used a seed-based method to generate the DMN and applied a receiver operator characteristic (ROC) analysis to evaluate the contribution of DMN connectivity strength. It was found that MPFC had the highest effectiveness (sensitivity = 81.5%, specificity = 70.4%)^27^; however, this accuracy rate was based on reclassification of the training set, rather than on cross-validation or classification of an independent test set. Our previous study^28^ used independent component analysis (ICA) to generate the DMN, DAN and ventral attention network (VAN) within each group and applied linear discriminant analysis to obtain a sensitivity of 90% and specificity of 81% using leave-one-out cross-validation (CV). The principal limitation of this work was that all features were preselected out of CV based on statistical analysis between groups. However, both of the previously discussed studies considered limited ICNs in the model generation, whereas the investigation of large-scale ICNs is more beneficial for understudying the neuropathological mechanism of MHE^22^.

There exists a vast body of literature regarding quantification of voxel-wise intrinsic connectivity levels of certain ICNs for MHE^20–23, 27, 28^. Most of these studies were based on general linear models (GLMs), a widely used method that models interactions by computing a t statistic between groups^29^. However, GLM-based methods are not designed to detect nonlinear associations^30^. To address this problem, we developed a set of algorithms that model interactions among brain regions and a clinical variable. This algorithm set, termed graphical model-based multivariate analysis (GAMMA), is a Bayesian approach to modeling structure-function and clinical variable associations^30–32^. GAMMA can detect complex nonlinear multivariate associations among image features and a clinical variable. GAMMA has been employed in many neuroscience related applications, such as brain volume morphometry of sickle cell disease^33^ and fMRI data of Alzheimer’s Disease^34^.

In this study, we test the hypothesis that ICN-related diagnostic models can accurately distinguish cirrhotic patients with MHE from those without MHE (NMHE) based on the GAMMA method and that the most representative ICN for MHE can be selected by machine learning methods. To the best of our knowledge, this is the first study to build large-scale ICN-related diagnostic models for MHE using a Bayesian approach (GAMMA). To test our hypothesis, we first generated twenty ICNs from resting-state fMRI for each subject based on dual regression^35^. We then applied a Bayesian-based method to identify the most predictable spatial patterns for each ICN^29^. Finally, machine learning algorithms were employed to determine the most predictive ICN for MHE based on accuracies, sensitivities and specificities.

## Results

### Demographic and clinical characteristics of subjects

Table 1 shows the demographic and clinical characteristics of subjects in the two groups. We found significant differences between groups on all neurocognitive tests and Child-Pugh scores but no significant differences between groups in terms of age, gender, education or previous history of overt HE.

**Table 1:** Demographic and clinical characteristics of subjects. Note: TMT-A, trail making test A; TMT-B, trail making test B; DST, digit-symbol test; BDT, block-design test. *p-*values marked with * were calculated by *χ* ^2^-test.

Characteristic	NMHE(n = 42)	MHE (*n* = 32)	*p*-value
Age (year)	49.9 ± 8.7	52.4 ± 9.3	0.23
Gender (Male/Female)	16/4	28/4	0.47*
Handedness (Right/Left)	42/0	32/0	1.00*
Education (years)	8.3 ± 2.5	7.6 ± 2.5	0.15
Etiology of cirrhosis (HBV/alcoholism/HBV+alcoholism/other)	33/7/1/2	25/0/4/3	—
Child–Pugh stage (A/B/C)	22/16/5	10/11/11	—
Child-Pugh Score	7.0 ± 2.0	8.2 ± 2.2	0.01
Previous history of overt HE (Yes/No)	10/32	11/21	0.32*
TMT-A (seconds)	47.3 ± 16.9	75.2 ± 19.8	<0.001
TMT-B (seconds)	124.1 ± 41.9	175.4 ± 47.6	<0.001
DST (raw score)	40.0 ± 11.0	25.1 ± 7.6	<0.001
BDT (raw score)	28.5 ± 9.6	18.5 ± 7.2	<0.001

### Predictive performances for each ICNs

**Figure 1:**
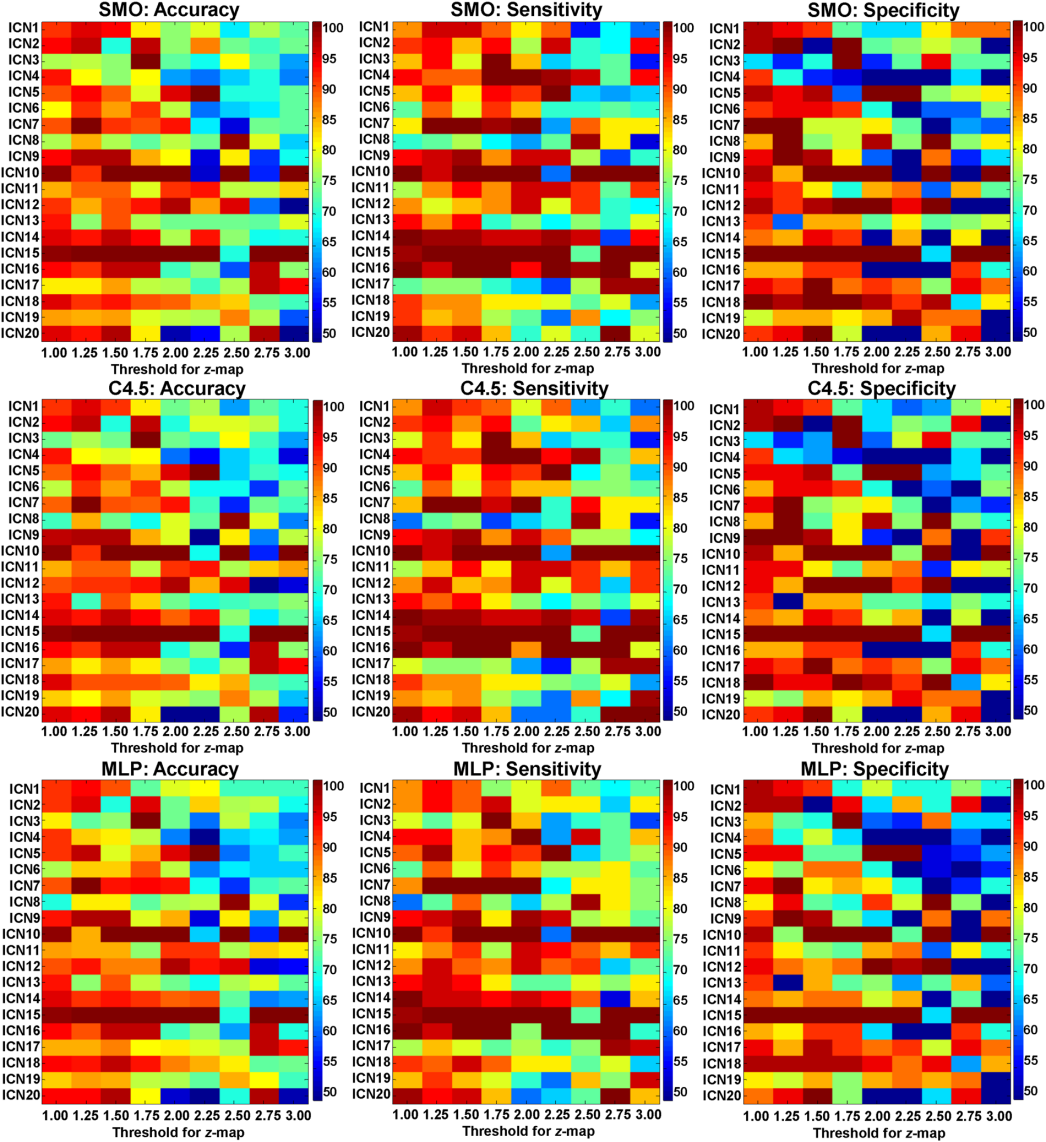
The predictive performances for all twenty ICNs based on GAMMA generating regional state variables at each stepwise threshold of the z-score maps. SMO: sequential minimal optimization; MLP: multilayer perceptrons.

Based on group-membership variable (MHE or NMHE), GAMMA generated the most predictive ROIs for each of the twenty ICNs. Figure 1 displays the predictive performance for the ROIs of all twenty ICNs at each stepwise threshold for the z-map. The performance of C4.5 was significantly better than that of multilayer perceptrons (MLP) in terms of sensitivity (MEAN_C4.5_ = 85.3 ± 12.1%, MEAN_MLP_ = 84.7 ± 12.4%, pairwise t-test, *p*_MLP/C4.5_ = 0.013). The performance of SMO were similar with the performances of C4.5 and MLP in regard to accuracy (Acc.), sensitivity (Sen.), and specificity (Spe.).

The 2 × 2 ANOVA results are provided in Table 2. From these results, it is evident that the thresholds and ICNs are the main effects for final performance. A significant interaction was identified between the thresholds and the ICNs for the final performance. The classifiers were not found to be a main effect for the final performance, and had no significant interaction between the thresholds or the ICNs.

**Table 2:** 2 × 2 ANOVA analysis for each of the two factors that influenced the final performances (Acc., Spe., and Sen.). THR: thresholds; CLS: classifiers; ICN: ICNs; df: degrees of freedom.

	*p*-values for Acc.	*p*-values for Spe.	*p*-values for Sen.
THR (df = 8)	**0**.**000**	**0**.**000**	**0**.**000**
CLS (df = 2)	0.977	0.915	0.851
ICN (df = 19)	**0**.**000**	**0**.**000**	**0**.**000**
THR × CLS (df = 16)	1.000	1.000	1.000
THR × ICN (df = 152)	**0**.**000**	**0**.**000**	**0**.**000**
CLS × ICN (df = 38)	1.000	1.000	1.000

The probability of obtaining accuracy above 85% among 9 thresholds and 3 classification methods was calculated for each ICN. The stable and representative ICNs were temporal lobe network (TLN, with a probability of 74.1% for Acc. > 85%), sub-cortical network (SCN, with a probability of 70.4% for Acc. > 85%), and sensorimotor network (SMN, with a probability of 88.9% for Acc. > 85%).

Performances were compared across all thresholds for the three ICNs. The best performance for TLN was at a threshold of 1.50, 1.75, 2.00, 2.50, and 3.00 (Acc. = 100%, Sen. = 100%, and Spe. = 100%); and SCN was at a threshold of 2.00 (Acc. = 97.4%, Sen. = 95.4%, and Spe. = 100%), and SMN was at a threshold of 1.25, 1.50, 1.75, 2.00, 2.25, 2.75, and 3.00 (Acc. = 100%, Sen. = 100%, and Spe. = 100%).

### Representative ICNs and their discriminative ROIs

**Figure 2:**
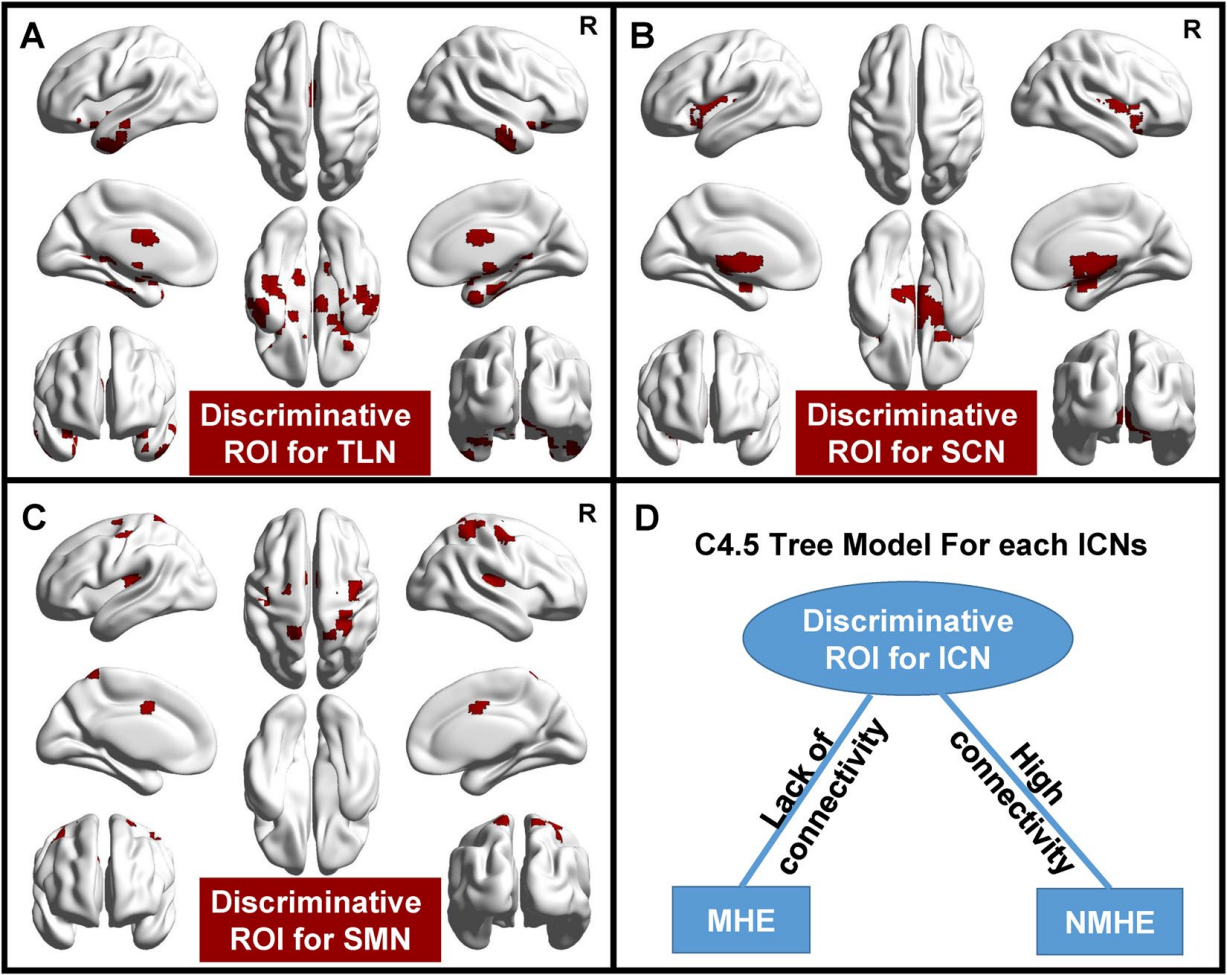
GAMMA-generating discriminative ROIs selected by the C4.5 tree model for the three ICNs (**A**–**C**), which can characterize group differences. C4.5 generates the same tree model for the three ICNs and selects only one ROI as predictive marker for discriminating MHE (**D**). The discriminative ROIs for each ICN were displayed by the BrainNet Viewer^66^ (Version 1.5; Beijing Normal University, Beijing, China, http://www.nitrc.org/projects/bnv/).

For each ICN, GAMMA generated a series of ROIs with a conditional probability table, from which the state of the condition (NMHE or MHE) can be inferred. According to the conditional probabilities, C4.5 selects the same tree model for the three ICNs (Fig. 2A–C) with the thresholds of the best performances. Only one ROI served as a predictive marker to discriminate MHE (Fig. 2D) in the tree model.

Table 3 shows that the two functional integration patterns of the discriminative ROIs within the ICNs were different between MHE and NMHE. The size of the discriminative ROIs within TLN, SCN, and SMN were markedly decreased in the MHE group. The average FC of the discriminative ROIs within TLN and SCN were significantly reduced in the MHE group.

**Table 3:** The group differences in size and average FC within discriminative ROIs for the most representative ICNs selected by C4.5 and GAMMA. Note: *p*-values < 0.05 are in bold type.

Name of ICN	TLN	SCN	SMN
Mean voxels of MHE in ROI	195 ± 75	448 ± 95	27 ± 13
Mean voxels of NMHE in ROI	270 ± 53	540 ± 88	38 ± 14
*p-*value for mean voxels	**0**.**000**	**0**.**000**	**0**.**001**
Mean FC of MHE in ROI	0.17 ± 0.38	2.09 ± 0.60	1.33 ± 0.96
Mean FC of NMHE in ROI	0.41 ± 0.33	2.53 ± 0.58	1.67 ± 0.92
*p*-value for mean FC	**0**.**005**	**0**.**002**	0.122
Threshold for *z*-map	1.75	2.00	2.75

### Relationship between functional integration patterns and clinical characteristics

**Figure 3:**
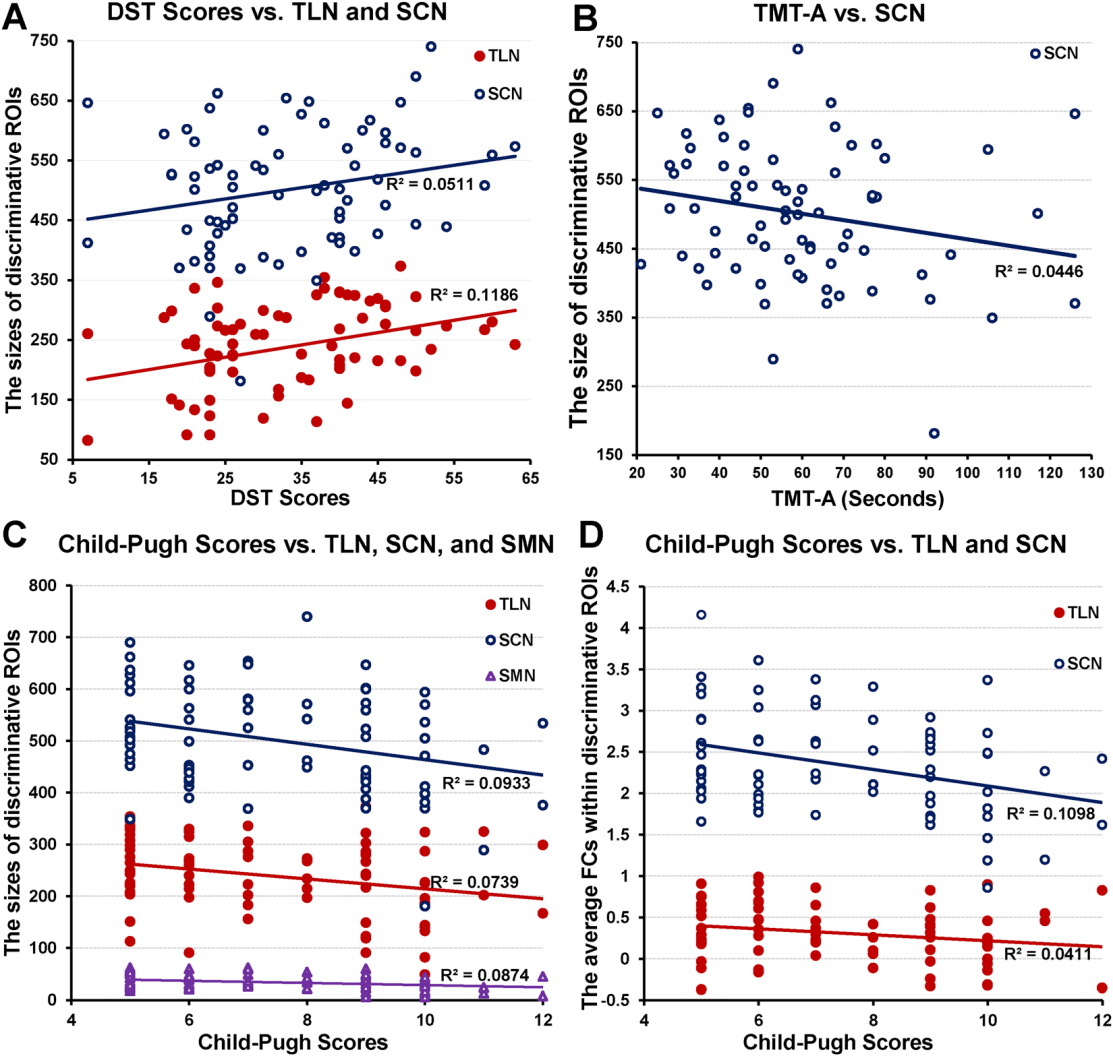
The significant relationships between the integration patterns of the ICNs and clinical characteristics. The relationship between the size of discriminative ROIs and DST Scores (**A**) TMT-A (**B**) and Child-Pugh Scores (**C**). The relationship between the average FCs within discriminative ROIs and Child-Pugh Scores (**D**).

Table 4 provides the correlation of functional integration patterns of discriminative ROIs within the three ICNs with clinical characteristics, using movement, age, gender and years of education as covariates. The significant *p*-values were corrected by the Bonferroni method. Figure 3 indicates that the DST scores were significantly positively correlated with both functional integrations of TLN. The BDT scores were significantly positively correlated with only one functional integration pattern (the size of discriminative ROIs) of TLN. The Child-Pugh scores were significantly negatively correlated with both functional integrations of TLN and SCN.

**Table 4:** The *p*-values for partial correlations between selected neuroimaging biomarkers and clinical criteria, using movement, age, gender and years of education as covariates. A. The size of discriminative ROIs; B. The average FC within discriminative ROIs. Note: *p*-values < 0.05 are in bold type, and *p*-values survived after Bonferroni corrected are marked with “*”.

ICN	TMT-A	TMT-B	DST	BDT	Child-Pugh scores
A. The size of discriminative ROIs for the most representative ICNs					
TLN	**0**.**040**	**0**.**027**	**0**.015*	0.075	**0**.000*
SCN	**0**.010*	0.131	**0**.006*	0.061	**0**.010*
SMN	0.466	**0**.**031**	0.212	0.371	**0**.012*
B. The average FC within discriminative ROIs for the most representative ICNs					
TLN	0.517	0.476	0.597	0.858	**0**.008*
SCN	0.088	0.480	0.061	0.119	**0**.006*
SMN	0.912	0.813	0.836	0.638	**0**.**044**

## Discussion

To the best of our knowledge, there has been no previous attempt to generate an ICN-related diagnostic model for MHE using the GAMMA approach. We found that the diagnostic models based on the three ICNs, TLN, SCN, and SMN, were accurate in distinguishing MHE patients from NMHE patients with high sensitivity and specificity. The GAMMA-based biomarkers selected by machine learning methods demonstrated that patients with MHE tended to have disconnections within these three ICNs, and distinct functional integration patterns were significantly associated with behavior criteria and Child-Pugh scores. This finding illustrates the utility of GAMMA-based ICN models for MHE identification.

The technical advantage of our study was the application of GAMMA for examining abnormalities of functional integration patterns in ICNs for the identification of MHE. GAMMA enabled us to identify distinct intrinsic connectivity regions strongly associated with MHE. Compared with the voxelwise analysis algorithms based on the general linear model, such as the voxelwise t test, GAMMA is nonparametric and makes no assumption about the probabilistic distribution between ICN connectivity and clinical variables. GAMMA is automatic; that is, it does not require the user to select parameters, such as the significance threshold required for a T-map. The GAMMA aims to maximize the Bayesian Dirichlet score among brain regions and the clinical variable *C* (which in this case was “MHE” or “NMHE”), instead of classification accuracy. The strength of GAMMA is dimension reduction, and GAMMA can parcellate the voxel space into brain regions. Therefore, GAMMA should not introduce significant bias in classification accuracy in cross-validation.

Moreover, three machine learning methods were applied to generate classifiers, in order to avoid bias with respect to the functional form of the classifier. SVM and MLP consider all input features in model generation and were less accurate than C4.5. These results suggest that an optimized feature selection strategy (C4.5) should be more available in our GAMMA-based model generation. Furthermore, the thresholds and ICNs exhibited main effects and significant interaction with the final performance (Table 2). The next strategy for the representative ICN selection was to consider the combination of the thresholds and ICNs. First, the three stable representative ICNs were selected because they had a probability above 70% for the Acc. > 85% among 9 thresholds and 3 classification methods. Second, the best performances for the three selected representative ICNs were obtained across 9 thresholds. Thus, the GAMMA-based models exhibited robustness and accuracy at certain thresholds for the representative ICNs.

It is important to note that we obtained more reliable and accurate performances than in our previous work^36^. However, the two studies were different in several regards. First, the aims of the two studies were different. In the present study, we generated predictive models for MHE from cirrhosis patients, and in the previous study, we generated models for MHE from healthy volunteers. Second, the methods of our previous work were different: (1) the functional connectivities between 116 pairs of brain regions based on an anatomical automatic labeling (AAL) template were calculated by Pearson correlation; (2) *p*-value ranking-based kernel principal component analysis (kPCA) was applied in the feature selection step to reduce the dimension of input data; and (3) we found that the FC-based diagnostic model was accurate in differentiating MHE from normal controls with 86.5% accuracy, 88% specificity and 85% sensitivity. Moreover, our GAMMA-based methods obtained better performance than previous MHE models based on DMN, DAN and VAN^27, 28^.

According to Fig. 2D, if the FCs within the discriminative ROIs are larger than the threshold (high connectivity within a certain ICN), the model considers the participants to be NMHE; otherwise, the model considers the participants to have MHE. Accordingly, the three ICNs based on the GAMMA method can be an accurate, objective and convenient tool for providing combinative and complementary information for the early diagnosis of the disease. Specifically, this method could be used in combination with neuropsychometric scales and computerized examinations, which are the current MHE diagnostic tools^37, 38^. Early diagnosis and subsequent treatment can modestly reduce the risk of cognitive dysfunction in MHE patients.

Generally, neuroimaging-based diagnostic models have been widely applied as complementary methods in diagnosing various neuro-related diseases and disorders, such as autism^24, 39^, Alzheimer disease^26, 40^, schizophrenia^41^, and attention-deficit/hyperactivity disorder^25, 42^.

Large-scale ICNs were involved in this study, providing an opportunity to organize the findings about altered networks into a systematical framework^43^. According to the performance of three machine learning methods, the most representative ICNs for MHE prediction were identified, which were TLN, SCN and SMN. The three predictive ICNs for MHE suggests that the GAMMA-based ICN models might provide new insight into cognitive abilities among cirrhotic patients.

We investigated the GAMMA-generated ROIs for MHE prediction by two functional integration patterns: (1) the size of discriminative ROIs, which indicated the spatial distributions of high functional connectivity within certain ICNs; and (2) the average FC within discriminative ROIs, which reflected the level of functional connectivity within certain ICNs. Both of the patterns indicated functional disconnections within these three ICNs, which suggests that the functional integration of the selected ICNs may be susceptible to impairments induced by liver decompensation.

Decreased functional connectivity of the TLN and SCN were observed in our study, and TLN and SCN were selected as the most representative ICNs with the discriminative ROI (Fig. 2A,B) for MHE. Accordingly, the cortical thickness of the superior temporal gyrus was found to be thinning in MHE patients^1^. In fMRI studies, the amplitude of low-frequency fluctuation (ALFF) of the middle temporal gyrus has been shown to decrease in MHE patients^15^. Similarly, a previous fMRI study reported changes in FC within the cortical-striatal circuits of cirrhotic patients^44^, and the gray matter volumes and density have been found to be significantly decreased within subcortical nuclei of cirrhosis patients^45–47^. However, impairments of the TLN and SCN have not been extensively studied, so our results may provide evidence for investigating the neuropathological mechanism of temporal-^48^ and subcortical-related^49^ memory deficits in MHE patients.

The fact that the SMN was selected as a representative ICN with the discriminative ROI (Fig. 2C) for MHE. The decreased sensorimotor-associated cortices are in line with previous MHE research: the regional homogeneity (ReHo)^14^ and ALFF^15^ were decreased within precentral gyrus and supplemental motor area of MHE patients. However, an ICA-based ICN study found that there was no significant differences in SMN between MHE and normal controls^22^. Thus, our dual-regression and GAMMA based methods can provide complementary information for the impairment of SMN, which may account for the motor dysfunction in MHE patients reported in the previous studies^50, 51^.

In general, our investigation of large-scale ICNs is beneficial for depicting the clinical state of MHE^22, 28^. Our ICN findings demonstrate that MHE is associated with aberrant connectivity patterns at the whole-brain scale^18, 43, 52, 53^. These abnormal connectivity patterns are critically involved in cognitive impairments, including problems with memory and motor functions^3–7, 50, 51^.

We found a positive correlation between DST scores and the functional integrations of TLN and SCN, and a negative correlation between TMT-A and the functional integrations of SCN. DST mainly focuses on the domains of memory, attention and psychomotor speed^9^, and TMT can provide information about visual search speed, scanning, speed of processing, mental flexibility and executive functioning^54^. Based on the functions of the TLN and SCN, the correlations with DST and TMT-A are quite expected. Moreover, Child-Pugh scores were negatively correlated with TLN, SCN, and SMN. The cognitive functions of vision, memory, and attention of MHE patients may be influenced by the level of liver decompensation (Child-Pugh scores)^55, 56^. Thus, the representative ICNs with discriminative ROIs based on GAMMA can serve as neuroimaging-based biomarkers providing supplementary information to be used with other existing MHE diagnostic methods.

One limitation of this study was using a relatively lower magnetic strength (1.5 T) and sampling rate (repetition time, 2.5 seconds) to obtain functional MR images, due to the finite capability of the MR scanner. Another limitation was the small sample size, although machine learning methods with cross validation were utilized. Therefore, we plan to recruit more participants and use a 3.0 T MR scanner to further investigate GAMMA-based MHE predictive models. Additionally, for a stringent and unbiased approach, future participants can be used as an independent test dataset to validate the detected biomarker and the predictive models. Furthermore, future studies will use an ICA driven approach in addition to our template matching methods, and a longitudinal study will be launched to validate and clarify the utility of the detected ICN-based biomarkers for detecting and monitoring MHE.

In conclusion, the three representative ICNs (TLN, SCN, and SMN) with discriminative ROIs for MHE were selected by machine learning methods from large-scale ICNs. The distinct functional integration patterns of the representative ICNs were significantly associated with behavior criteria and Child-Pugh scores. Our findings suggest that the representative ICNs with discriminative ROIs based on GAMMA could be potential neuroimaging biomarkers for MHE identification, and provide supplementary information to other existing MHE diagnostic methods.

## Materials and Methods

### Subjects

The Institutional Ethics Committee of Zhongda Hospital, Medical School of Southeast University (Nanjing, China) approved all experimental procedures in accordance with the 1964 Declaration of Helsinki and its later amendments or comparable ethical standards. All participants gave written informed consent before MR imaging or neuropsychologic evaluation.

Thirty-three cirrhotic patients with MHE and forty-three cirrhotic patients without MHE (NMHE) were enrolled after written consent. General information about the subjects is summarized in Table 1. Subjects were excluded in the case of neurological or psychiatric diseases, decompensate diabetes, renal failure, and severe cardiovascular disease. Additional exclusion criteria included individuals who were taking psychotropic medications, or had abused alcohol within 6 months prior to the study.

MHE was diagnosed by neuropsychiatric tests, including Trail Making Test A (TMT-A), Trail Making Test B (TMT-B), Digit Symbol Test (DST), and Block Design Test (BDT). DST and BDT are subtests of the Wechsler Adult Intelligence Scale-Revised for China (WAIS-RC). MHE was defined as being impaired two standard deviations beyond normative performance on at least two of four tests (i.e., TMT-A > 68 seconds, TMT-B > 156 seconds, raw DST score < 23, or raw BDT score < 16). The normative values for the local population were determined from a sample of 160 healthy controls that were age and education matched to the patients in this study.

### Data Acquisition

MRI data were collected using a 1.5 T scanner (Vantage Atlas; TOSHIBA, Nasu, Japan). The participants were instructed to rest with their eyes closed, not to think of anything in particular, and keep their head still during fMRI scanning. Functional images were collected with an echo planar imaging sequence (TR/TE = 2500 ms/40 ms, FOV = 24 cm × 24 cm, matrix = 64 × 64, FA = 90°, slice thickness/gap = 5 mm/1 mm, 22 axial slices) to measure 120 brain volumes. High-resolution, three-dimensional T1-weighted images were also obtained with following parameters: 108 sagittal slices, FOV = 256 mm × 256 mm, matrix = 256 × 256, slice thickness/gap = 1.5 mm/0 mm. MR images were reviewed for quality by an experienced radiologist. We excluded subjects with poor MR image quality.

### Preprocessing of resting-state fMRI

**Figure 4:**
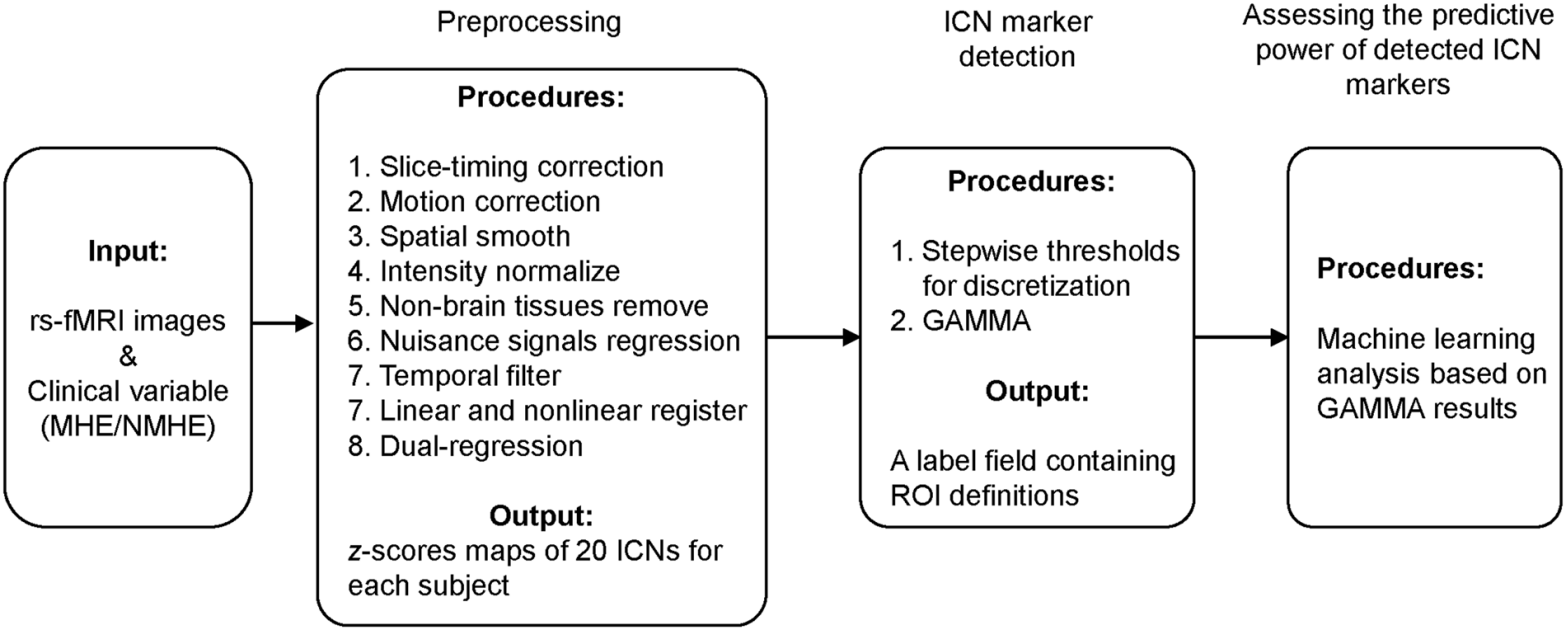
The data processing pipelines.

The data processing pipelines are displayed in Fig. 4.

fMRI data processing was carried out using FSL (Version 5.0; FMRIB Software Library, Oxford, UK, www.fmrib.ox.ac.uk/fsl)^57, 58^ and AFNI (Version 11.12.21.1014; NIH, Maryland, USA, afni.nimh.nih.gov)^59^, according to Hallquist *et al*. ^60^ as follows: discarded first five volumes; slice-timing correction using Fourier-space time-series phase-shifting; motion correction using MCFLIRT; mean-based intensity normalization of all volumes by the same factor; removal of non-brain tissue; regressing out nuisance signals (i.e., Friston-24 motion parameters, CSF, whiter matter, global signal as well as linear and quantic trends) based on 3dDeconvolve; band-pass temporal filtering (0.01 −0.1 Hz); spatial smoothing using a Gaussian kernel of FWHM 6 mm. After preprocessing the fMRI volumes were registered to the subject’s high-resolution T1-weighted scan using affine registration (FLIRT), and subsequently to standard space (MNI152) images using nonlinear registration (FNIRT) with a spatial resolution of 3 mm × 3 mm × 3 mm.

### ICN generation

**Figure 5:**
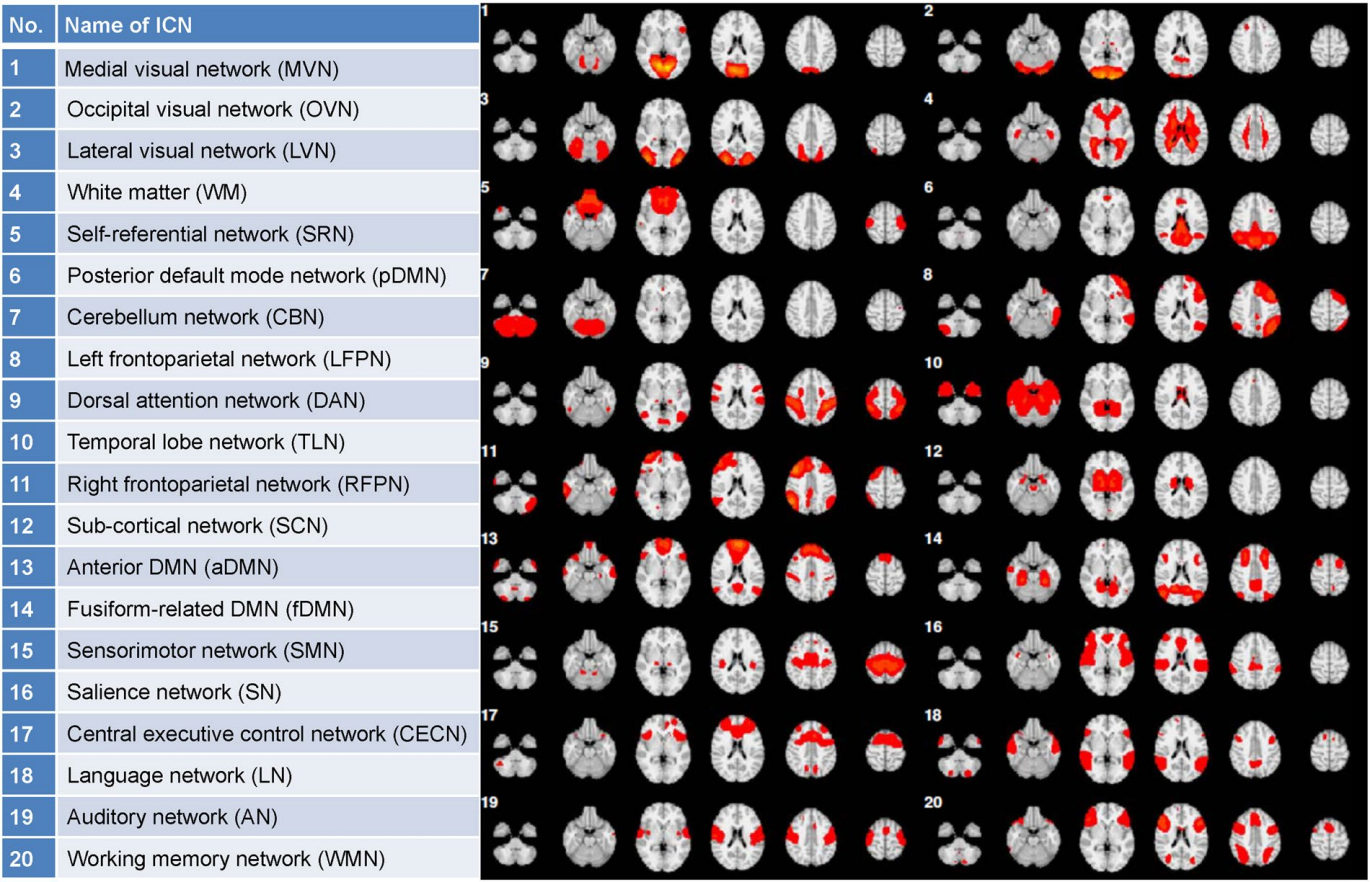
Name and anatomical structure of ICNs.

For each individual, dual regression was applied to generate twenty most representative ICNs, the standard templates of which were obtained from the 1000 Functional Connectomes Project (http://fcon_1000.projects.nitrc.org)^35^. The dual-regression analysis contains two steps: (1) spatial regression and (2) temporal regression (for details, please see ref. 61). First, a set of twenty template ICNs were used as regressors in a spatial multiple regression conducted on the 4d functional volumes for each subject. This resulted in an individual time course of an array of beta values, each of which represents the spatial pattern of activity for the corresponding ICN at each time point^61^. Second, the individual time courses were used as regressors in a temporal regression conducted on each voxel of the 4d functional volumes for each subject, resulting in an individual 3d ICN map for each subject^61^. Finally, twenty individual ICNs for each subject were obtained after the entire dual regression process. For detailed information regarding the twenty template ICNs, see Fig. 5 and previous research^35^.

The mean spatial maps for all subjects were transformed to *z*-scores for display. The *z*-scores used here reflect the degree to which a given voxel’s time series correlates with the time series corresponding to a specific ICN template, scaled by the standard deviation of the error term^62^. Therefore, the *z*-scores could be used to measure how much of the standard deviation of the signal was from background noise, i.e., the *z*-scores represented the intensity of resting activity for each subject in the ICN studies.

### Graphical model-based multivariate analysis (GAMMA)

To identify abnormal integration patterns for intrinsic connectivity, we discretized certain ICN *z*-score maps (normal distribution: mean = 0, and standard deviation = 1), which were generated from dual regression. For the stability of the GAMMA model, we set 9 voxelwise thresholds for *z*-score maps from 1 to 3 (step value was set as 0.25). So, for subject *i*, if voxel *X* was larger than the threshold, we labeled this voxel “1” (high connectivity within a certain ICN); otherwise, we labeled it “0” (lack of connectivity in the ICN). The result of the discretization step for subject *i* was a high connectivity map ${D}^{i}$.

Next, GAMMA was applied to automatically detect the representative voxels and generate the ROI for each representative voxel. Additionally, an embedded model validation step was used to determine whether the model could be a statistical artifact. Finally, the output of the GAMMA was a conditional probability table, which is able to infer the state of the condition (NMHE or MHE) for each particular ROI of a certain ICN. Therefore, the ROI state variables can be used as features for the predictive model to identify the predictive powers for the ROIs of a certain ICN. Further details regarding the GAMMA can be found in Supplementary 1 and previous articles^30, 32^.

### Machine learning algorithms

To avoid algorithm bias, we applied several commonly used machine learning methods, including support vector machines (SVMs), multilayer perceptrons (MLP), and C4.5, to identify the predictive powers of the ROIs. The three machine learning methods were implemented in WEKA (Version 3.6; The University of Waikato, Hamilton, New Zealand), which is a widely used machine learning software package, and the default parameters in WEKA were applied for all three methods.

For SVM, a sequential minimal optimization (SMO) with polynomial kernel was applied, which is a fast iterative algorithm to efficiently solve the optimization problem in SVMs^63^. It divides the optimization problem of SVMs into a set of smallest possible subproblems, which are then solved analytically. SMO is most effective for linear SVMs, and can significantly improve scaling and computation time of SVMs.

The MLP maps inputs onto a set of appropriate outputs using a feed forward artificial neural-network model^64^. An MLP uses three or more layers of nodes with nonlinear activation functions, and thus, is more powerful than the standard linear perceptron for modeling nonlinear decision boundaries. MLP parameters are typically optimized based on internal cross-validation; hence, we also optimized MLP parameters using this approach.

C4.5 is an extension of the ID3 algorithm. C4.5 can generate a pruned or unpruned decision tree^65^. At each node of the tree, C4.5 chooses the attribute of the data that most effectively splits its set of samples into subsets enriched in one class or the other. The splitting criterion is the normalized information gain (difference in entropy). The attribute with the highest normalized information gain is selected to make the decision. The C4.5 algorithm then recurs on the smaller sub lists.

The predictive powers of ROIs for certain ICNs were evaluated based on 10-fold cross-validation, and the performance of the predictive powers were evaluated based on the following metrics: sensitivity (Sen.), specificity (Spe.), and accuracy (Acc.).

### Statistical Analysis

The software SPSS Statistics (Version 21.0; IBM, Chicago, USA) was used for statistical analysis.

To evaluate the performance of the three machine learning methods, we first averaged performances (accuracy, sensitivity, and specificity) across thresholds for each ICN, and then we compared average performance of the 20 ICNs between each machine learning method using pairwise t-tests. A *p* value < 0.05 was considered to indicate a significant difference.

There were three factors that could influence performance: thresholds, types of classifiers, and types of ICNs. As a result, 2 × 2 ANOVAs were applied for detecting main effects and significant interactions that could influence the final performance (Acc., Spe., and Sen.). A *p* value < 0.05 was considered to indicate a main factor or significant interaction for the performances.

Next, the probability for the accuracy above 85% among 9 thresholds and 3 classification methods was calculated for each ICN. Finally, the ROIs for certain ICNs with probabilities of 70% were selected as the stable predictive models to discriminate MHE patients from NMHE patients. The selected ROIs for the ICNs with the best performance among nine thresholds were considered as the discriminative ROIs of the most representative ICNs for MHE.

### Evaluation of GAMMA Generated Biomarkers

GAMMA-generating discriminative ROIs of the most representative ICNs were evaluated in the following steps.

Let ${{ROI}}^{j}({{ICN}}^{k})$ denote the *j*th ROI for the *k*th ICN, which was selected by C4.5. Let ${D}^{i}({{ICN}}^{k})$ represent the high connectivity discretization map for subject *i* of the *k*th ICN, and ${D}^{i}{({ICN}}^{k})\cap {{ROI}}^{j}({{ICN}}^{k})$ represents the overlap ROI for ${{ROI}}^{j}({{ICN}}^{k})$ and ${D}^{i}$. As a result, ${N}_{{D}^{i}{({ICN}}^{k})\cap {{ROI}}^{j}({{ICN}}^{k})}$ represents the number of voxels that were in both ${{ROI}}^{j}({{ICN}}^{k})$ and ${D}^{i}$. Let ${F}^{i}({{ICN}}^{k})$ represent the functional connectivity map for subject *i* of the *k*th ICN, and ${{FC}}_{{F}^{i}({{ICN}}^{k})\cap {{ROI}}^{j}({{ICN}}^{k})}$ represents the average function connectivity for subject *i* within the *j*th ROI of the *k*th ICN. Both ${N}_{{D}^{i}{({ICN}}^{k})\cap {{ROI}}^{j}({{ICN}}^{k})}$ and ${{FC}}_{{F}^{i}({{ICN}}^{k})\cap {{ROI}}^{j}({{ICN}}^{k})}$ were two functional integration patterns of the *j*th ROI for the *k*th ICN. The two-sample t-test was applied to calculate different sizes (${N}_{{D}^{i}{({ICN}}^{k})\cap {{ROI}}^{j}({{ICN}}^{k})}$) and FCs (${{FC}}_{{F}^{i}({{ICN}}^{k})\cap {{ROI}}^{j}({{ICN}}^{k})}$) of discriminative ROIs between MHE and NMHE. A *p* value < 0.05 was considered to indicate a significant difference.

To investigate the relationship between discriminant results and behavior criteria, we computed correlation coefficients between the GAMMA results and Trail Making Test A (TMT-A), Trail Making Test B (TMT-B), Digit Symbol Test (DST), Block Design Test (BDT), and Child-Pugh scores. In this study, we computed partial correlations between two functional integration patterns of the discriminative ROIs within the selected ICNs and clinical criteria by controlling for movement (the motion parameters were optimized by the band-pass temporal filter according to the previous study^60^), age, gender and years of education. The *p*-value < 0.05 (Bonferroni corrected) was considered as a significant correlation.

## Electronic supplementary material

Supplementary 1
