# Supplementary material for: Predictive models of minimal hepatic encephalopathy for cirrhotic patients based on large-scale brain intrinsic connectivity networks
Source: Sci Rep. 2017 Sep 14;7:11512. doi: 10.1038/s41598-017-11196-y (PMC5599725; doi:10.1038/s41598-017-11196-y)
Supplement: Supplementary file 1 — Supplementary 1 [file 41598_2017_11196_MOESM1_ESM.pdf]

Predictive models of minimal hepatic encephalopathy for cirrhotic patients based on large-scale brain intrinsic connectivity networks

Yun Jiao[1], Xun-Heng Wang[2], Rong Chen[3], Tian-Yu Tang[1], Xi-Qi Zhu[1, 4], Gao-Jun Teng[1]*

[1] Jiangsu Key Laboratory of Molecular and Functional Imaging, Department of Radiology, Zhongda Hospital, Medical School of Southeast University, Nanjing 210009, China

[2] College of Life Information Science and Instrument Engineering, Hangzhou Dianzi University, Hangzhou 310018, China

[3] Department of Diagnostic Radiology and Nuclear Medicine, University of Maryland, School of Medicine, Baltimore, MD 21201, USA

[4] Department of Radiology, The Second Hospital of Nanjing, Medical School of Southeast University, Nanjing 210003, China

* Email: gjteng@vip.sina.com

Supplementary 1:

GAMMA protocols

The input to GAMMA was the collection of difference maps and a clinical variable *C*. *C* was a categorical variable that represents group membership [1]; in this study, *C* represented either MHE or NMHE. Regions of interest (ROIs) that were predictive of *C* could be identified using GAMMA. These ROIs can be used as the neuroanatomical markers of *C*.

GAMMA then detected a set of brain regions that were highly predictive of *C*. However, because our analysis was at the voxel level, brain regions were not predefined. Therefore, GAMMA also needed to group voxels into regions. The steps that were used to identify brain regions characterizing group differences and to group voxels into regions were referred to as representative voxel detection and brain region delineation, respectively [2,3].

GAMMA performed representative voxel detection and brain region delineation in an iterative fashion. For representative voxel detection, it searched representative voxels using forward selection. $\mathbf{RV}$ denoted the set of representative voxels. Initially, $\mathbf{RV}$ was empty, and the search space for representative voxels, [VS], contained all voxels. In iteration k, $\mathbf{RV}$ contained $k-1$ representative voxels, and $\mathbf{RV}=\{\mathrm{RV}_1,...,\mathrm{RV}_i,...,\mathrm{RV}_{k-1}\}$. For the voxels in [VS], we identified one voxel that could generally improve the predictive power of the representative voxel set when added to $\mathbf{RV}$. The predictive power of $\mathbf{RV}$ was quantified based on Bayesian Dirichlet Equivalent score. Then, we added this voxel RV_k to RV. If no such voxel existed, GAMMA stopped [2,4].

For brain region delineation, in iteration k, after identifying a representative voxel RV_k, we searched [VS] to identify voxels that were probabilistically equivalent to RV_k, i.e., for

which the probabilistic association between the voxel and *C* was similar to that of RV_k and *C*. We used a belief map learning algorithm to solve this problem. RV_k and voxels that were probabilistically equivalent to RV_k consist of a single ROI in the label field [5].

Finally, the output of GAMMA was a conditional probability table indicating the state (non-MHE or MHE) for each particular ROI of certain ICNs.

References

1 Chen, R. & Herskovits, E. H. Graphical-Model-based Morphometric Analysis. *IEEE Trans Med Imaging* **24**, 1237-1248 (2005).

2 Chen, R. & Herskovits, E. H. Graphical-model-based multivariate analysis of functional magnetic-resonance data. *Neuroimage* **35**, 635-647 (2007).

3 Chen, R., Hillis, A. E., Pawlak, M. & Herskovits, E. H. Voxelwise Bayesian lesion-deficit analysis. *Neuroimage* **40**, 1633-1642 (2008).

4 Chen, R. *et al.* Brain morphometry and intelligence quotient measurements in children with sickle cell disease. *J Dev Behav Pediatr* **30**, 509-517 (2009).

5 Chen, R. & Herskovits, E. H. Graphical model based multivariate analysis (GAMMA): an open-source, cross-platform neuroimaging data analysis software package. *Neuroinformatics* **10**, 119-127 (2012).
